# P-1715. Antibiotic Stewardship based on the usage of Non- FDA Approved Fixed Dose Combination of Antibiotics in a Secondary Care Hospital South India

**DOI:** 10.1093/ofid/ofae631.1880

**Published:** 2025-01-29

**Authors:** C Nitheeshwar, Suresh Kumar Dorairajan

**Affiliations:** Jaya college of paramedical science, chennai, Tamil Nadu, India; Apollo hospital, Chennai, Tamil Nadu, India

## Abstract

**Background:**

In developing countries, the widespread use of non-FDA (Food and Drug Administration) approved fixed-dose combinations of antibiotics in secondary care hospitals is a concern. Despite cautions from infectious disease specialists, comprehensive studies on this practice are lacking. Our research aims to investigate indications for prescribing these combinations in a South Indian secondary care hospital and develop stewardship strategies to reduce the use of it.

**TABLE 1:**
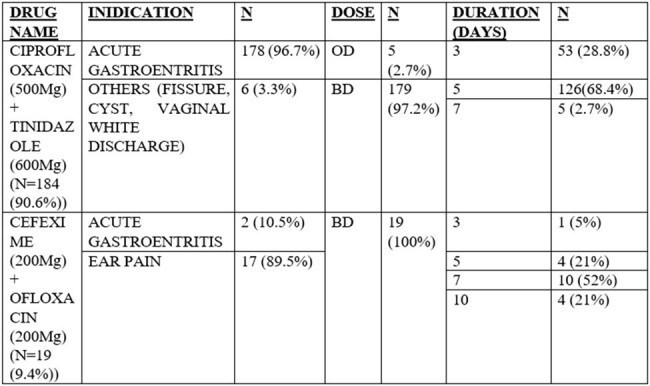
Indication for the Usage of non FDA Approved Fixed Combination of Antibiotics

**Methods:**

The study conducted was a Prospective observational study at a secondary care hospital in South India over a 4-month period from December 2023 to March 2024. Data collection utilized a validated audit tool capturing details of non-FDA approved fixed dose antibiotic combinations prescribed to patients. Statistical analysis was conducted using MS Excel, employing simple descriptive statistics.

**Results:**

CIPROFLOXACIN (500mg) + TINIDAZOLE (600mg) and CEFEXIME (200mg) + OFLOXACIN (200mg) are commonly prescribed for various conditions such as acute gastroenteritis, ear pain, and other ailments like fissures, cysts, and vaginal white discharge. However, these combinations lack FDA approval and randomized controlled trials (RCTs) to support their efficacy in treating conditions like otitis media and loose stools. The addition of antiparasitic agents in these combinations lacks evidence of effectiveness. Therefore, caution is advised in their prescription until further scientific validation is available.

**Conclusion:**

In conclusion, our antibiotic stewardship efforts led to the removal of CIPROFLOXACIN (500mg) + TINIDAZOLE (600mg) and CEFEXIME (200mg) + OFLOXACIN (200mg) from our hospital formulary. Physicians were informed that these combinations lack scientific rationale for treating specific conditions. This action aligns with evidence-based prescribing practices to optimize patient care and combat antimicrobial resistance.

**Disclosures:**

**All Authors**: No reported disclosures

